# Validity of a Commercially Available Inertial Measurement Unit for Artificial Intelligence-Based Trick Detection and Kinematic Performance Assessment in Skateboarding

**DOI:** 10.3390/s26082537

**Published:** 2026-04-20

**Authors:** Birte Scholz, Niklas Noth, Maren Witt, Olaf Ueberschär

**Affiliations:** 1 Institute for General Training and Movement Sciences, Faculty of Sports Science, Leipzig University, 04109 Leipzig, Germany; mwitt@uni-leipzig.de; 2 Department of Engineering and Industrial Design, Magdeburg-Stendal University of Applied Sciences, 39110 Magdeburg, Germany; 3 Institute for Applied Training Science, 04109 Leipzig, Germany

**Keywords:** skateboarding, freestyle sports, performance, kinematic analysis, training monitoring, validation study, motion capture, artificial intelligence, technology

## Abstract

Inertial measurement units (IMUs) present promising avenues for performance diagnostics in skateboarding, yet systematic validation of their accuracy and applicability remains limited. This study validates the commercially available Spinnax Freak IMU system in the context of skateboarding, with a focus on selected trick detection and classification, distance measurement, maximal horizontal speed, maximal vertical height of the skateboard and airtime during a jump trick. A total of 23 skateboarders (4 females, 19 males; 27.4 ± 10.9 years) participated in this study. Validation methods included comparisons with established reference systems such as laser ranging for maximal horizontal speed (LAVEG), 2D video analysis for maximal vertical height of the skateboard (Kinovea), light barrier measurements for airtime detection (OptoJump Next), and a fixed metric reference (10 m) for rolling distance measurements. The evaluation was supported by statistical analyses including mean absolute error (MAE), root mean-square error (RMSE), mean absolute percentage error (MAPE), *t*-tests, Bland–Altman plots, linear regression, and ICC(3,1). The Spinnax Freak system demonstrated high validity in detecting trick events and in providing distance measurements that were statistically equivalent to the reference. Trick classification, maximal horizontal speed, maximal vertical height of the skateboard and airtime showed substantial errors, indicating that these outputs are not reliable for biomechanical interpretation at this point. These findings highlight both the potential and the current constraints of single-sensor setups for field-based motion capture in skateboarding. Future developments should prioritize algorithmic refinement, improved temporal resolution, and optimized event classification to enhance measurement accuracy and expand applicability in biomechanical analysis and automated training documentation in skateboarding.

## 1. Introduction

### 1.1. Background

With the admission of skateboarding as an Olympic sport in 2017, the sport is only at the beginning of systematic scientific support structures in the field of sports science [1]. The disciplines Park and Street were first introduced at the Tokyo 2021 Olympic Games and were contested again at Paris 2024 [2]. The International Olympic Committee has announced that skateboarding will be included in the 2028 Los Angeles Olympics as a fully fledged, permanent Olympic sport [3]. These developments increase the demand for objective, reproducible methodologies to quantify and assess training, with the ultimate goal of optimizing competitive performance and injury risks in skateboarding [4].

The disciplines Park and Street require athletes to perform complex tricks in specifically designed environments. Tricks are acrobatic movements performed by riders to demonstrate technical skill and control [5]. While Park features flowing transitions and vertical elements, and Street includes rails, ledges, and stairs, both formats center on the execution of aerial and technical tricks [6]. In both disciplines, difficulty, variety, execution, use of the course, ‘flow’ (fluidity and expression), and consistency of the tricks are assessed by judges in accordance with the pertinent criteria of the international organization World Skate [7]. In terms of biomechanical metrics, the parameters maximal horizontal speed, distance, airtime, and maximal vertical height of the skateboard during a jump trick play an important role in the judging of trick execution quality [7]. From the standpoint of exercise science, systematically monitoring the number and type of tricks performed during training appears promising for targeting the judging criteria of variety in contests, as such data-driven feedback enables the adaptation of practice to increase the likelihood of successfully landing a broader range of tricks in competition. To objectively capture and analyze such kinematic performance metrics during training, inertial measurement units (IMUs) offer a field-based and data-driven approach with the potential to support training by enabling real-time quantification of trick performance and systematic documentation of executed tricks. In recent years, IMUs have become a robust alternative to optical motion-capture systems, as they are unaffected by common limitations such as occlusion, restricted capture volumes, or sensitivity to lighting conditions, while offering high portability and enabling reliable kinematic data collection in unconstrained, real-world environments [8,9,10]. Artificial intelligence enables the automated detection, classification, and interpretation of complex trick patterns. This makes it possible to provide IMU-based feedback to help athletes improve key judging dimensions during contest preparation, including the variety of performed tricks, repetition count within the performance, and quality of trick execution through objective data on movement patterns and trick sequences.

The Spinnax Freak (SF) is a commercially available motion-capture sensor specifically designed for skateboarding. This validation focuses on selected kinematic metrics provided by the SF system, including airtime, maximal vertical height of the skateboard during a jump trick, maximal horizontal speed, and distance covered, which are compared against reference measurements. Furthermore, the accuracy of an AI-based trick classification of two flat-ground tricks, i.e., Ollie and Kickflip, is validated. As an independent assessment of a commercially available IMU system and its proprietary AI-generated outputs (black-box), this study does not propose a new algorithm but instead examines the ability of the system to detect events and quantify movement parameters, thereby identifying both strengths and limitations relevant for applied use. These findings may inform future applications of IMU-based feedback in skateboarding for training, coaching, and competition preparation, particularly in disciplines where objective movement analysis is currently underdeveloped.

The rest of this article is organized by first outlining the related previous research that frames the study, followed by a detailed description of the materials and methods used for data acquisition and processing. The subsequent parts present the experimental results, discuss their implications in the context of existing research, and conclude with a conclusion of the main findings and future research directions. This study aims to evaluate the validity of the outputs from the SF sensor system and its associated smartphone application in detecting and quantifying skateboarding tricks and movement parameters under field conditions.

### 1.2. Related Previous Research

AI-based algorithms for movement classification rely on machine learning (ML) and, in parts, deep learning (DL) methods that automatically learn discriminative patterns from sensor data rather than requiring manually engineered features. In IMU-based skateboard trick classification, related studies have used a broad range of machine learning models, including principal component analysis combined with support vector machines (PCA and SVMs), k-nearest neighbors (kNNs), random forests (RFs), decision trees (DTs), naïve Bayes (NB), logistic regression (LR), rule-based Partial Decision Rule (PART) models, Gaussian process classification (GPC), and k-means clustering, as well as deep learning approaches such as artificial neural networks (ANNs), multi-layer feedforward neural networks (MFFNNs), one-dimensional convolutional neural network U-Net architectures (1D-CNN U-Net), transfer learning models such as MobileNet, MobileNetV2, NasNet, ResNet101 and ResNet101V2, and heuristic peak-based event detection methods. Prior work on sport-specific IMU-based, AI-enhanced movement identification has demonstrated compelling classification accuracy for trick detection in acrobatic sports and board sports, including artistic skating [11], cheerleading [12] and snowboarding [13,14]. Several studies have also reported automated flat-ground trick detection in skateboarding with high accuracy levels [15,16,17,18,19,20,21]. Depending on the type of skateboarding trick performed, the AI-based algorithm used, and the number of trials, trick classification accuracies reported in the literature range from 89.1% to 100.0%. For example, Corrêa et al. [16] reported an accuracy of 98.7%, while Abdullah et al. [17] achieved up to 95.0%, Groh et al. [19] documented values of up to 89.1%, and Abdullah et al. [18] reached 100.0%. A comprehensive overview of all included IMU-based trick-classification studies, their methodological characteristics, sensor configurations, sample sizes, and reported performance metrics is provided in Table 1, offering a structured comparison of the current state of research across skateboarding, snowboarding, artistic skating, and cheerleading.

Despite this methodological progress, to date, only one study has investigated a test–retest reliability of a skateboard-mounted sensor for trick detection. It was based on data collected from a single participant only [22]. That study found no significant differences between repeated trials for each trick, reporting high reliability indicators across all metrics (ICC  >  0.80, Cronbach’s *α* > 0.80, *r*  >  0.80; *p*  <  0.001). These findings suggest that an IMU-based setup is a reliable tool for capturing skateboarding trick performance and may serve as a methodological reference for validating future custom-built sensor systems. According to the extant literature, no studies have systematically validated the performance of IMUs in detecting or quantifying skateboarding tricks and movement parameters.

## 2. Materials and Methods

### 2.1. Participants

Participants were recruited through the German Roller Sports and Inline Skating Association (Deutscher Rollsport- und Inline-Verband e.V., Frankfurt am Main, Germany; DRIV) and local skateboarding communities in Leipzig, Germany. A total of 23 skateboarders (4 females, 19 males) participated in the study and the cohort represents a convenience sample. The mean age of the participants was 27.4 ± 10.9 years (range: 8–53), the mean stature was 1.76 ± 0.16 m (1.26–1.95 m), the mean body mass index (BMI) of the participating adults (21–53 years) was 23.1 ± 2.5 kg m^−2^ (mean ± standard deviation; range 18.6–28.7 kg m^−2^), while it amounted to 17.0 ± 0.8 kg/m^2^ (range: 16.2–18.3 kg m^−2^) for the children (8–13 years). Median BMI values corresponded to the percentile 37.5 ± 12.5 of the national German reference data according to Kromeyer-Hauschild et al. [23]. Participants had been skateboarding for an average of 11 ± 7.9 (3–31) years and trained on average 5.8 ± 3.8 (0.5–12.0) hours per week. The stance distribution comprised 15 regular (left foot at the front) and 8 goofy (right foot in the front) skateboarders. Inclusion criteria were defined as (1) being healthy and athletically active, (2) possessing at least two years of consistent skateboarding experience, and (3) being able to perform a set of predefined tricks and skills (e.g., 1. Stable stance on a skateboard; 2. Stable riding a skateboard; 3. Ollie) reliably. Exclusion criteria encompassed any cardiopulmonary diseases, acute musculoskeletal injuries, infectious conditions or any other physical impairments that could impede informed participation or reliable data acquisition. All participants were briefed orally and in writing regarding the purpose and procedures of the study. All participating minors provided written guardian consent and were accompanied and supervised during data collection by a legal guardian and the regional coach for the State of Saxony from DRIV.

### 2.2. Equipment

**Figure 1:**
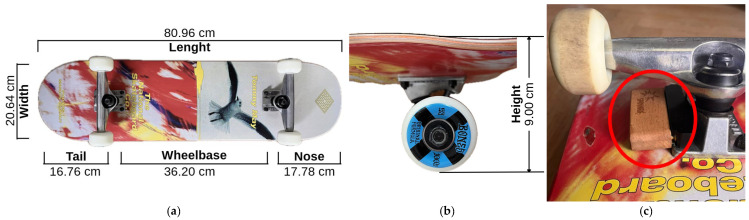
Technical dimensions of the skateboard deck used in this study were taken (**a**) from manufacturer specifications (width, length, tail, wheelbase, and nose [25]) and (**b**) from self-measurements the complete skateboard setup height including wheels, trucks, and skateboard deck. (**c**) Employed SF inertial measurement unit (highlighted by red circle) mounted beneath the skateboard deck, positioned on the trucks.

The structural setup of a typical skateboard comprises the four fundamental elements, deck, trucks, wheels, and bearings, each fulfilling distinct mechanical functions essential to its performance [6]. For the present study, a skateboard deck measuring 8.125″ (20.64 cm) in width and 31.875″ (80.96 cm) in length, with a 14.25″ (36.20 cm) wheelbase, a 7″ (17.78 cm) nose, and a 6.6″ (16.76 cm) tail, was employed. As for the wheels, Bones 100s-OG #4 V5 with a diameter of 53 mm and a hardness rating of 100A were utilized. In addition, tensor Alloys 5.5″ regular trucks were used, featuring a hanger width of 140 mm, an axle width of 8.125″, and a mid-height of 53 mm. The selection of this standardized design ensures geometric consistency and practical relevance, as it reflects the prevalent configuration utilized in contemporary Street and Park skateboarding disciplines [6]. Figure 1a,b illustrate the technical dimensions of the skateboard setup used in this study.

The SF sensor system (v1.2.0, Spinnax GmbH, Bodman-Ludwigshafen, Germany) is an IMU designed for skateboard-specific motion tracking. Mounted beneath the skateboard deck, the device connects wirelessly via Bluetooth to a dedicated smartphone application, enabling the recording of skateboarding sessions and the real-time processing of motion data through integrated artificial intelligence (AI)-based algorithms. The system is intended to automatically detect and identify performed tricks and provide corresponding kinematic parameters, such as airtime and maximal vertical height of the skateboard during a jump trick. In addition, the application provides further spatiotemporal metrics including distance covered and maximal horizontal speed throughout the session. It integrates triaxial accelerometers (tri-axial acceleration ${\vec{a}=\left(a_{x},a_{y},a_{z}\right)}^{T},\;\left|a_{x_{i}}\right|\leq \pm 8\;g$), gyroscopes (tri-axial angular velocity ${\vec{\omega }=\left(\omega_{x},\omega_{y},\omega_{z}\right)}^{T},\;\left|\omega_{x_{i}}\right|\leq \pm 2000\;{}^{\circ}s^{-1}$), and magnetometers (800 μT) [24]. The sampling rate and internal filtering parameters of the SF system were not disclosed by the manufacturer and therefore remain unknown. The SF sensor is mounted beneath the skateboard deck, positioned directly on the trucks using a custom spacer (Figure 1c). It was installed according to the manufacturer’s instructions, remained firmly secured throughout all trials, and did not shift or change position at any point during data collection. The sensor connects to the SF smartphone app via Bluetooth. In order to facilitate continuous data storage, the sensor needs to permanently remain within the Bluetooth range throughout the measurement.

The OptoJump Next system (Microgate Srl, Bolzano, Italy) was used as a reference to assess spatiotemporal parameters of locomotion and jump performance through the concept of light barrier arrays. OptoJump Next consists of transmitter and receiver LED gate arrays that detect interruptions in infrared light beams, allowing precise calculation of contact time, airtime, and jump height. It has been widely validated and employed in sport science, e.g., for airtime [26,27], jump height [26,28] or sprint performance [29] diagnostics. Furthermore, the laser-based LAVEG system (LDM301A, ASTECH GmbH, Rostock, Germany) was employed in this study to record the distance between a reference point and the moving subject, allowing indirect calculation of maximal horizontal speed along the straight riding path based on temporal changes in position, with signals sampled at a frequency of 100 Hz [30,31]. Video recordings were obtained with a consumer digital camera at a resolution of 1920 × 1080 pixels and 50 fps (LEGRIA HF G30, Canon Inc., Ōta, Tokyo, Japan). For distance validation, a manually marked 10 m reference track was established using a standard 50 m measuring tape.

### 2.3. Study Design

**Figure 2:**
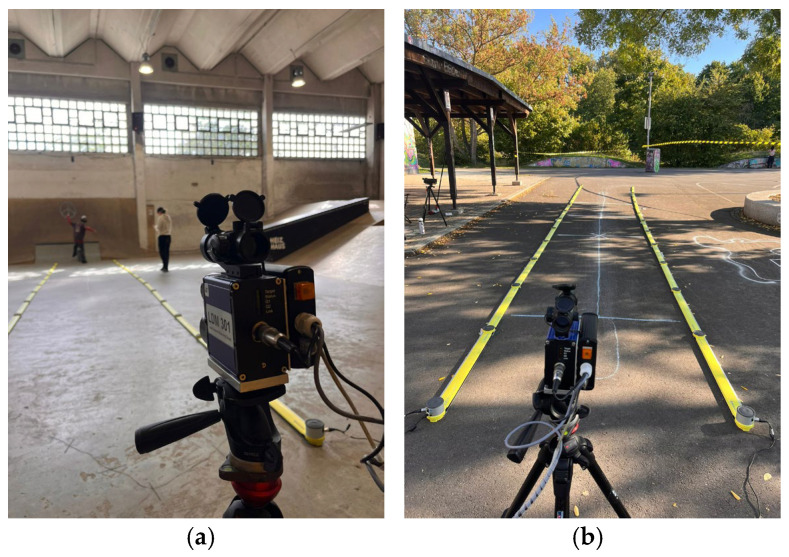
Experimental setup in (**a**) the indoor, and (**b**) the outdoor setting, showing the LAVEG system in front and the OptoJump Next gates in the background.

All measurements were conducted within a one-week period in September 2025 at the Heizhaus skating facility in Leipzig, Germany, utilizing both the indoor hall and the adjacent outdoor skatepark depending on weather conditions. The combination of indoor and outdoor settings was chosen purposely to best reflect the variety of ambient conditions under which the SF is intended to operate. The digital video camera was positioned laterally at the take-off zone, with the optical axis aligned perpendicular to the movement direction to minimize potential parallax errors. Participants were instructed to ride along a clearly marked straight line and to keep the skateboard parallel to this line to ensure a consistent perspective for height estimation. The LAVEG system was aligned along a straight riding path, targeting the participants’ lower back to approximate the position of the center of mass and ensure consistent distance tracking. The OptoJump Next system was installed over a 13 m segment indoors, and an 8 m segment outdoors, the latter due to practical spatial constraints. Lateral panel spacing was 191 cm indoors and 161 cm outdoors. These variations were acceptable within the scope of the present validation study, as all participants had sufficient space in both environments to perform the required tricks without restriction. In Figure 2, the experimental setup with LAVEG and OptoJump Next is shown for both indoor and outdoor conditions.

Table 2 provides an overview of the output metrics generated by the SF app for skateboarding performance analysis. Each metric is defined and linked to its corresponding reference system used for validation in this study.

**Figure 3:**
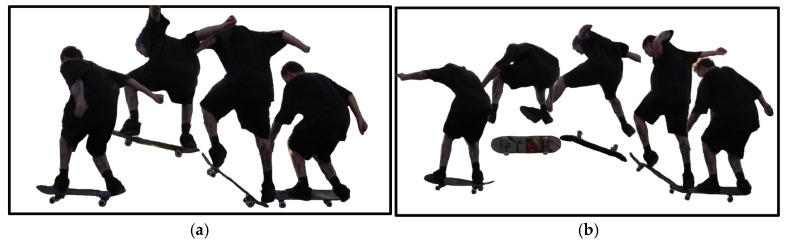
Example of a movement sequence (with horizontally stretched time axis) of (**a**) an Ollie and (**b**) a Kickflip performed by a study subject moving from right to left.

The experimental procedure began with a five-minute self-directed warm-up session within the skatepark. This phase primarily served to allow participants to physically prepare and to familiarize themselves with the provided experimental setup, ensuring consistent equipment handling across trials. Following the warm-up, each participant performed five Ollies and, if feasible, five Kickflips. The Ollie, as a foundational trick, involves coordinated foot separation and board rotation around the rear wheels to achieve lift-off without hand contact [32]. Biomechanically, it consists of five distinct phases, with successful execution depending on timing, board geometry, and precise foot movements to reach sufficient height and horizontal alignment [32,33] (Figure 3a). The Kickflip is a fundamental aerial trick in skateboarding, executed by initiating a 360-degree rotation of the board around its longitudinal [34]. This is achieved by the rider performing an Ollie while simultaneously dragging the front foot diagonally off the edge of the board, generating the necessary torque for the flip (Figure 3b).

For each trick, the following parameters were recorded: airtime (s) and maximal vertical height of the skateboard (cm). Trick detection was defined as a binary variable (true/false), indicating whether a performed trick was successfully detected. Trick classification was defined as a categorical variable with two possible outcomes per measurement series: Ollie or none in the Ollie trials, and Kickflip or none in the Kickflip trials. Here, ‘none’ denotes that the detected event did not correspond to the target trick of the respective series. If the trick was not successfully landed, the attempt was repeated until a complete execution was achieved and could be reliably documented, or the subject aborted the trial. All documented tricks were therefore successfully landed.

**Figure 4:**
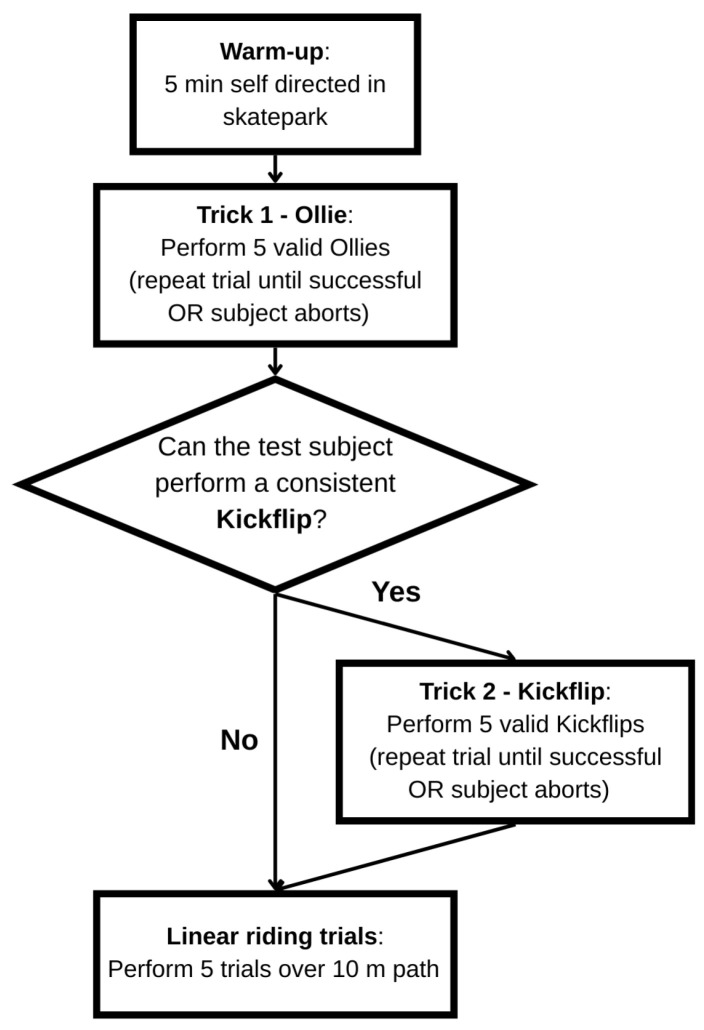
Flow chart of measurement procedure.

Subsequently, participants completed five linear riding trials over a predefined 10 m path. A manually marked 10 m reference track was used to validate the total distance covered. The start and end points were defined using a measuring tape and highly visible marking lines on the floor with a thickness of ~1 cm. The measuring tape had an accuracy of ±0.5 mm (corresponding to half a scale unit), implying that the resulting maximal systematic measurement error was dominated by the marking line thickness and can be considered negligible in the context at hand, amounting to ±2⋅1 cm ≘ 0.2%. To minimize any potential onset of fatigue, participants experienced a brief measurement-specific pause between trials due to the required data-saving process, and the executed skateboard tricks themselves were technically demanding but not muscularly strenuous, making relevant fatigue effects unlikely [35]. Figure 4 presents the flow diagram illustrating the entire measurement procedure.

### 2.4. Data Processing

All data were processed using Microsoft Excel (v2108) and Python (v3.10). For every documented trick, the app-generated outputs from the SF system including airtime, maximal vertical height of the skateboard during a jump trick, maximal horizontal speed, distance covered, trick detection, and trick classification were reviewed directly within the smartphone application. Each trick was checked individually, and the corresponding data were manually transferred into a structured spreadsheet. Synchronization between the IMU outputs and the reference systems (OptoJump, video, LAVEG) was achieved using a combined mechanical-visual trigger and the validated internal clocks of the devices. This approach allowed a precise and unambiguous matching of each IMU output to its corresponding reference trial, ensuring accurate trial-level alignment despite the IMU providing only processed event-based data. Only successfully landed tricks with complete sensor outputs were included. Trick detection was binary-coded: if a trick was successfully identified/classified by the system, a value of 1 was assigned; if not, a value of 0 was recorded.

Raw distance data from the LAVEG system were checked for measurement artifacts in terms of outliers (i.e., implausible sudden changes $\left|d_{i}-d_{i-1}\right|>\;$0.5 m between consecutive time steps *i* and *i* + 1 with Δt = 10 ms at 100 Hz) and absolute values exceeding a defined upper limit of 10 m. Invalid or missing values were then linearly interpolated. The curated distance values were filtered to retain only those between 1 m and 10 m. Horizontal speed was calculated using the five-point central difference method [36]:

(1) $$v_{i}=\frac{-d_{i+2}+8d_{i+1}-8d_{i-1}+d_{i-2}}{12\Delta t};\;\;d_{-1}=d_{-2}=0$$

To further reduce noise, a moving average filter with a window of 10 data points (100 ms) was applied [30]:

(2) $$v_{smooted,i}=\;\frac{1}{10}\sum_{j=i-4}^{i+5}v_{j};\;\;v_{j}=0\;for\;j<0.$$

Finally, smoothed horizontal speed values outside the plausible range of 0–20 km h^−1^ were excluded. The resulting dataset included time, cleaned distance, and smoothed maximal horizontal speed values.

The maximal vertical height of the skateboard per jump trick was analyzed using the video analysis software Kinovea (version 2023.1.2) [37]. The distance between the ground and the skateboard deck at the height of the wheel (9.00 cm) visible in each video was used for vertical calibration. All participants performed their tricks in the same designated area, ensuring consistent calibration across trials [38]. The camera was positioned at a fixed lateral angle and remained stable throughout all recordings to maintain uniform scaling conditions. The final maximal vertical height of the skateboard was derived from pixel-based measurements and systematically documented in a results spreadsheet for subsequent analysis.

The Airtime data were recorded using the proprietary software of OptoJump Next (version 1.14.11.0) and exported for each participant. Maximal airtime values were manually transferred into a structured results spreadsheet.

### 2.5. Statistical Analysis

All statistical analyses were conducted using Python (v3.10). Detection recall or precision were calculated separately for Ollies and Kickflips, distinguishing between general trick detection and trick-specific classification. For the kinematic parameters, mean values and standard deviation were computed for each measurement system. Boxplots were included as complementary visualizations, as they illustrate median, IQR, and outliers, offering distributional insights that supplement the mean-based statistical analyses. Mean absolute error (MAE), root mean squared error (RMSE), and mean absolute percentage error (MAPE) were calculated to quantify differences between the SF and the reference systems. Equivalence of the distance measurement was assessed using two one-sided *t*-tests (TOSTs), with a predefined margin of ±0.5 m. Equivalence margin was defined a priori to represent the maximal deviation considered acceptable for practical application in the given sport-specific framework. The choice of margin was guided by the specific measurement context, balancing statistical rigor with ecological validity: the margin was set small enough to detect meaningful systematic differences, yet wide enough to account for natural variability in human movement and measurement noise. For all remaining comparisons, paired *t*-tests were conducted. Specifically, a two-sided paired *t*-test was used to compare maximal horizontal speed between systems. For maximal vertical height of the skateboard, a one-sided paired *t*-test was applied based on prior evidence that the video system consistently measured higher values than SF. For airtime, a one-sided paired *t*-test was conducted because SF consistently measured longer airtime than OptoJump Next. For all paired *t*-tests, Cohen’s *d* was calculated as an effect size and interpreted according to Cohen [39] as follows: *d* > 0.2: small effect, *d* > 0.5: medium effect, *d* > 0.8: large effect. Agreement was further assessed using Bland–Altman analysis, which provided estimates of systematic bias and the 95% limits of agreement (LoA = bias ± 1.96 SD) [40]. A regression analysis was performed within the Bland–Altman plot to assess potential proportional bias between the two measurement systems. Within-subject reliability across repeated trials was evaluated using the intraclass correlation coefficient ICC(3,1). For variables used in the reliability analysis, missing values were handled using mean imputation when the proportion of missing data was sufficiently small (0.0–15.0%) [41]. ICC values are interpreted such that values below 0.5 indicate poor reliability, values between 0.5 and 0.75 indicate moderate reliability, values between 0.75 and 0.9 indicate good reliability, and values above 0.9 indicate excellent reliability [42]. Statistical significance was defined as *p* < 0.05 for all hypothesis tests. Prior to all parametric tests, normality was assessed using the Kolmogorov–Smirnov test applied to participant-level mean values, and all aggregated data met the assumption of normality.

## 3. Results

Table 3 summarizes the number of valid and missing trials for each metric and outlines the corresponding practical error tolerance, providing a transparent overview of the dataset prior to the detailed statistical results. Missing values occurred when the SF system did not generate an output for a given trial, for example due to incomplete event detection. Additional missing values resulted from the fact that not all participants were able to complete the full set of planned trials within the available time, which reduced the number of valid observations for specific metrics. The practical error tolerances were selected to reflect the magnitude of measurement error that would still allow meaningful interpretation of the metrics in applied skateboarding coaching and performance monitoring.

### 3.1. Trick Detection and Classification

A total of 19 participants performed Ollie attempts during the detection trials. The SF system was used to detect whether or not a trick was detected per attempt, regardless of the specific trick type. Across all trials, 87 Ollies were executed. The SF system detected a (i.e., any) trick in 83 of these attempts, while 4 Ollies were not detected, resulting in a recall of 95.4% for Ollie detection. In the subsequent classification analysis, the system correctly identified 51 of the 83 detected tricks as Ollies, whereas 32 detected tricks were misclassified, yielding a precision of 61.4% for Ollie classification.

**Figure 5:**
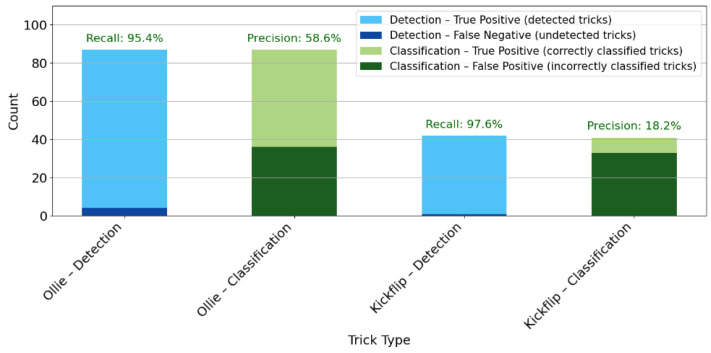
Detection (recall) and classification (precision) metrics for Ollie and Kickflip tricks, combining specific counts, percentage recall and percentage precision.

Furthermore, a total of 12 participants were able to perform the Kickflip and thus took part in the corresponding subsequent detection trials. Across these trials, 42 Kickflip attempts were executed. The SF system detected a trick in 41 cases, corresponding to a recall of 97.6% for Kickflip detection. Out of the 41 tricks detected, 8 were correctly classified as Kickflips, while 33 were misclassified, resulting in a precision of 19.5% for Kickflip identification. Figure 5 illustrates the detection and classification performance of the SF system for Ollie and Kickflip tricks, displaying the number of successfully detected and correctly classified attempts for each trick type, alongside the corresponding recall or precisions.

### 3.2. Total Horizontal Distance

**Figure 6:**
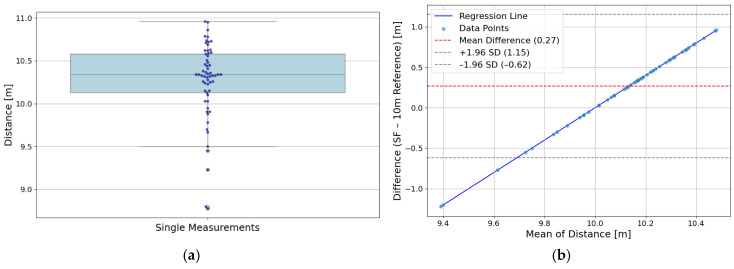
(**a**) Boxplot with swarm plot of all SF distance measurements across all trials. (**b**) Bland–Altman plot comparing SF distance measurements to the exact reference value of 10.00 m, showing bias (0.27 m; red dashed line), limits of agreement (−0.62 m, 1.15 m; gray dashed lines), and a linear regression (blue solid line).

A total of 61 trials were recorded from 14 participants, each completing the predefined 10 m distance between two and five times. The distances measured by the SF app were compared to the known, exact reference value of 10.00 m. For each participant, the mean value of all recorded trials was calculated individually. Based on all trials, the overall average distance measured by the SF app was 10.27 ± 0.45 m, resulting in an MAE of 0.45 m, an MAPE of 4.5% and a RMSE of 0.52 m. Figure 6a shows a boxplot with overlaid swarm plot visualizing the distribution of all SF distance measurements.

To evaluate the accuracy of the SF apps’ distance measurement, a statistical equivalence test was conducted using two one-sided testing (TOST). An equivalence margin of ±0.5 m was defined a priori, reflecting the acceptable range of deviation for practical applications in skateboard-specific exercise science. The TOST for yielded equivalence (*p* < 0.02; lower bound: mean ≥ 9.5 m, *p* < 0.001; upper bound: mean ≤ 10.5 m, *p* < 0.0192). Figure 6b displays a Bland–Altman plot comparing SF distance measurements with the 10 m reference. The intraclass correlation coefficient ICC(3,1) was calculated based on five repeated measurements per subject. After row-wise mean imputation of missing values (12.9%), the ICC(3,1) yielded a point estimate of 0.56, with a 95% confidence interval ranging from 0.22 to 0.73.

### 3.3. Maximal Horizontal Speed

**Figure 7:**
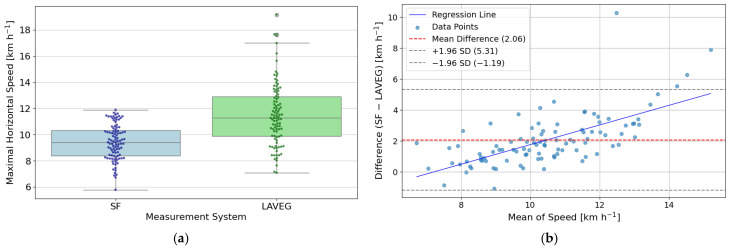
(**a**) Boxplots with swarm plots of SF and LAVEG maximal horizontal speed measurements across all trials. (**b**) Bland–Altman plot comparing SF and LAVEG maximal horizontal speed measurements, showing bias (2.06 km h^−1^; red dashed line) and limits of agreement (−1.19 km h^−1^, 5.31 km h^−1^; gray dashed lines) with a linear regression (blue solid line).

The analysis of maximal horizontal speed included data from 23 subjects, comprising a total of 99 paired trials recorded simultaneously with the SF and LAVEG measurement systems. Across all subjects, the SF system recorded a mean maximal horizontal speed of 9.46 ± 1.34 km h^−1^, while the LAVEG system yielded a mean of 11.60 ± 2.32 km h^−1^. The overall MAE between SF and LAVEG was 2.08 km h^−1^, with an MAPE of 17.0% and an RMSE of 2.25 km h^−1^. Figure 7a shows a boxplot with overlaid data points comparing the distribution of maximal horizontal speed measurements between the SF and LAVEG systems. A paired two-sided *t*-test revealed a statistically significant difference between SF and LAVEG (*T* = 12.37, *p* < 0.001, *d* = 1.24), confirming that LAVEG consistently measured higher speeds than SF. Figure 7b presents a Bland–Altman plot comparing SF and LAVEG maximal horizontal speed measurements, revealing a mean difference (i.e., bias) of 2.06 km h^−1^ between LAVEG and SF maximal horizontal speed measurements. The standard deviation of the differences was 1.66 km h^−1^. Based on this, the 95% limits of agreement were calculated as −1.19 km h^−1^ (lower limit) and 5.31 km h^−1^ (upper limit). The ICC(3,1) for absolute agreement between SF and LAVEG was 0.7015, indicating moderate agreement. Missing data were handled using mean imputation (10.0%). The 95% confidence interval ranged from 0.41 to 0.86, and the result was statistically significant (*p* < 0.001).

### 3.4. Maximal Vertical Height of the Skateboard During Jump Trick

**Figure 8:**
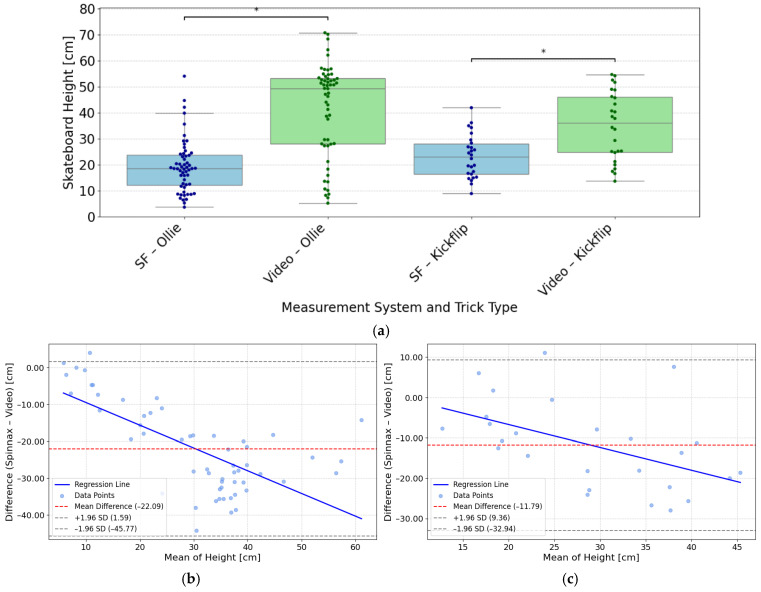
(**a**) Boxplots and swarm plots of SF and video-based maximal vertical skateboard heights during airtime across all trials, separated by trick type (Ollie and Kickflip). * Indicates significance at *p* < 0.001 (one-sided paired *t*-test). (**b**,**c**) Bland–Altman plot comparing SF and Video maximal vertical skateboard height measurements (**b**) of the Ollie, showing bias (−22.09 cm; red dashed line) and limits of agreement (−45.77 cm, 1.59 cm; gray dashed lines) with a linear regression (blue solid line) and (**c**) of the Kickflip, showing bias (−11.79 cm; red dashed line) and limits of agreement (−32.94 cm, 9.36 cm; gray dashed lines) with a linear regression (blue solid line).

During the Ollie trials, the SF application reported 56 valid measurements for the maximal vertical height of the skateboard during airtime. The reported mean was 19.31 ± 10.21 cm, whereas video analysis using Kinovea indicated a substantially higher mean maximal height of 41.39 ± 17.95 cm. For the Kickflip trials, 26 valid measurements were obtained. For those, the SF system reported a mean Kickflip maximal vertical skateboard height during airtime of 23.09 ± 8.33 cm, as compared to 34.87 ± 13.18 cm retrieved by video-based Kinovea analysis. Figure 8a shows a boxplot comparison of maximal vertical skateboard height measurements for Ollie and Kickflip tricks during airtime, as obtained from SF and through video analysis. The plot highlights differences in median heights, variability, and individual data points between both systems and trick types.

Error metrics revealed larger discrepancies for Ollie compared to Kickflip. Specifically, the MAE was 22.27 cm for Ollie and 13.83 cm for Kickflip. The RMSE was 25.12 cm for Ollie and 15.84 cm for Kickflip. The MAPE was 51.1% for Ollie and 38.6% for Kickflip. Based on prior evidence that video consistently measured higher values than SF, a one-sided paired *t*-test was conducted. For Ollie trials, video measurements were significantly higher than SF (*T* = −13.68, *p* < 0.001, *d* = 1.83), and for Kickflip trials likewise (*T* = −5.57, *p* < 0.001, *d* = 1.09). These findings confirm that the video system reported greater maximal vertical heights across both trick types. Figure 8b shows the difference in Ollie maximal vertical skateboard height measurements between SF and video analysis plotted against their mean. The red dashed line marks a bias of −22.09 cm, with limits of agreement at −45.77 cm and 1.59 cm. A blue regression line indicates proportional bias. Figure 8c depicts the difference in Kickflip maximal vertical skateboard height measurements between SF and video analysis plotted against their mean. The red dashed line indicates a bias of −11.79 cm, with limits of agreement at −32.94 cm and 9.36 cm.

The ICC was not computed for skateboard height because the proportion of imputed values was too high to ensure a reliable agreement estimate [41], which resulted from the fact that SF only recorded a height when a trick was successfully detected.

### 3.5. Airtime

**Figure 9:**
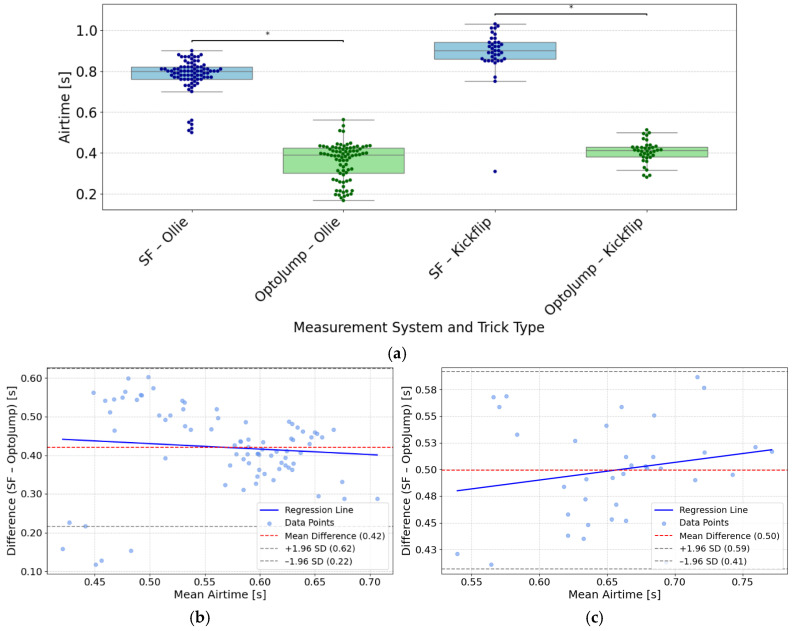
(**a**) Boxplot and swarm plot of SF and OptoJump Next airtime measurements across all trials, separated by trick type (Ollie and Kickflip). * Indicates significance at *p* < 0.001 (one-sided paired *t*-test). (**b**,**c**) Bland–Altman plot comparing SF and OptoJump Next airtime measurements (**b**) of the Ollie, showing bias (−0.42 s; red dashed line) and limits of agreement (0.22 s, 0.62 s; gray dashed lines) with a linear regression (blue solid line) and (**c**) of the Kickflip, showing bias (−0.50 s; red dashed line) and limits of agreement (0.41 s, 0.59 s; gray dashed lines) with a linear regression (blue solid line).

For the analysis of airtime during Ollie attempts, data from 81 valid paired measurements were included. The SF system for the Ollie reported a mean airtime of 0.78 ± 0.08 s, while the OptoJump Next system measured a mean airtime of 0.36 ± 0.09 s. For the analysis of airtime during Kickflip, data from 37 valid paired measurements were included. The SF system for the Kickflip reported a mean airtime of 0.89 ± 0.12 s and the OptoJump Next system a mean airtime of 0.41 ± 0.06 s. Figure 9a shows a boxplot comparison of airtime measurements for Ollie and Kickflip tricks, recorded by SF and OptoJump Next. For the Ollie, the MAE was 0.42 s, the RMSE was 0.43 s, and the MAPE between the two systems amounted to 133.4%. For the Kickflip the MAE was 0.49 s, the RMSE was 0.50 s and MAPE was 122.0%. Because SF consistently measured longer airtime than OptoJump Next, a one-sided paired *t*-test was conducted. Airtime was significantly higher for SF in both Ollie trials (*T* = 36.19, *p* < 0.001, *d* = 4.02) and Kickflip trials (*T* = 27.66, *p* < 0.001, *d* = 4.55). Figure 9b compares the difference in Ollie airtime measurements between SF and OptoJump Next analysis plotted against their mean. The red dashed line indicates a bias of 0.42 s, with limits of agreement at 0.22 s and 0.62 s. A blue regression line indicates proportional bias. Figure 9c illustrates the differences in Kickflip airtime measuerments between SF and OptoJump Next relative to their mean.

The mean bias is 0.50 s, with limits of agreement at 0.41 s and 0.59 s. The calculated ICC(3,1) for Ollie Airtime was 0.09 (0.01 to 0.24) and for Kickflip Airtime was −0.0 (0.0, 0.1). Mean imputation was applied to replace missing airtime values (13.8%).

## 4. Discussion

This study aimed to evaluate the detection performance, trick-specific classification accuracy, and the measurement validity of key performance metrics, specifically maximal horizontal speed, maximal vertical height of the skateboard, and airtime, by comparing the SF system with reference methods. Nevertheless, the lack of transparency regarding the undisclosed internal algorithms and calculation procedures should be acknowledged when interpreting the results. Because the internal signal-processing and machine-learning procedures of the SF system are not disclosed, the present study evaluates the performance of the complete integrated system. To avoid conflating different error sources, we distinguish between (i) algorithm-related performance (detection and classification) and (ii) the measurement validity of continuous metrics, which reflects the accuracy of the sensor–processing pipeline rather than the classification model. This distinction is essential because classification errors arise from the machine-learning model, whereas measurement deviations in distance, speed, height and airtime originate from the sensor–processing pipeline.

### 4.1. Algorithm Performance: Trick Detection and Classification

The system demonstrated high detection performance (95–98% recall), whereas its ability to correctly classify the specific trick type was substantially lower (20–59% precision). This contrast highlights a central limitation: while general movement patterns are captured robustly by SF, the finer distinctions between tricks remain challenging. Ollies showed moderate classification success, suggesting that their signal features are somewhat distinct but still prone to overlap with other movements. Kickflips, in contrast, were rarely identified correctly, which points to the complexity and variability of this trick. Rapid board rotation and diverse execution styles likely produce heterogeneous sensor signals that the current model cannot consistently separate. This issue reflects a typical challenge in the classification of more complex tricks, where high variability and limited training data lead to class imbalance and reduced model performance [43]. It is possible that overlapping features between different tricks blur the decision boundaries, making classification more difficult. The limited amount of Kickflip training data and the resulting class imbalance could also have influenced the model performance. In addition, variations in how participants executed the tricks may have reduced within-class consistency, which might explain the lower accuracy observed. The heterogeneity of the sample, including both children and adults, reflects the typical composition of national and international skateboarding cohorts, where competitive athletes are characteristically young and training groups span a wide age range. This diversity increases variability in execution styles but enhances ecological validity by representing real-world users of such systems. Taken together, these findings suggest that the system is well-suited for detecting trick occurrence but requires refinement for reliable trick-specific classification. Improvements in feature engineering, balanced datasets, and personalized calibration could enhance accuracy, especially for complex tricks like Kickflips. Particularly in skateboarding, where movement dynamics are complex and execution styles vary widely, single-sensor setups may lack the resolution needed for precise biomechanical analysis. Future developments may incorporate custom IMU data processing pipelines, including resampling, segmentation, windowing, and normalization, to enable interpretable feature extraction for trick classification.

### 4.2. Hardware–Algorithm Performance: Kinematic Measurement Validity

Before interpreting the continuous metrics, it is important to note that height and airtime are only computed after the system has detected a trick sequence. Although this creates a functional dependency on the detection algorithm, these variables represent continuous measurement outputs derived from the sensor–processing pipeline rather than from the machine-learning classification model. In contrast, distance and speed are recorded continuously and independently of trick detection.

The SF system demonstrated solid performance in measuring predefined distances, with an average deviation of 0.27 m from the exact 10.00 m reference and a MAPE of 4.5%. These results suggest that the system is well-suited for estimating short linear distances in applied skateboarding-specific exercise science contexts, which is particularly beneficial for training documentation and for determining the actual distance covered during a competition run. The slight overestimation of the 10.00 m reference aligns with the biomechanical tendency of participants to roll marginally beyond the target due to horizontal momentum. The statistical equivalence test confirmed that the measurements fall within an acceptable range for practical use. While the ICC value (0.56) indicates moderate consistency across repeated trials, this may reflect natural variation in movement execution or minor environmental influences. It is possible that slight deviations in skating paths, sensor placement, or surface conditions contributed to the observed variability. Overall, the findings point to a promising level of accuracy.

The SF system showed moderate agreement with the LAVEG reference in measuring maximal horizontal speed, with an average deviation of 2.06 km h^−1^ and a relative error of 17.0%. While the ICC value of 0.70 indicates acceptable consistency across trials, the paired two-sided *t*-test revealed a statistically significant difference between SF and LAVEG. This suggests that the SF system systematically underestimates maximal horizontal speed compared to the reference, exhibiting a relative error that increases at higher velocities. The Bland–Altman analysis supports this interpretation, showing a consistent bias and wide limits of agreement. The regression line in the Bland–Altman plot further indicates a proportional bias, with larger discrepancies occurring at higher horizontal speeds. One possible explanation for this trend could be the filtering algorithm used by the SF app, which may smooth peak values and thereby dampen higher maximal horizontal speed readings. Differences in measurement principles, sampling rates, or response timing might also contribute. Although the lower back and the skateboard represent different points along the kinematic chain, and therefore naturally yield slightly different measurement values, any resulting systematic deviation can be considered negligible due to the predominantly linear movement pattern. Overall, the systematic underestimation and wide limits of agreement indicate that these estimates should be interpreted with caution, particularly when precise quantification of maximal speed is required. Enhancing temporal resolution, optimizing peak detection algorithms, and calibrating against dynamic reference standards could help reduce systematic deviations.

The SF system consistently reported lower maximal vertical skateboard heights during airtime compared to video analysis, with larger discrepancies observed for Ollie than for Kickflip. This systematic underestimation was confirmed by the paired *t*-test, which revealed a statistically significant difference between the two measurement methods. The Bland–Altman plots for both Ollie and Kickflip show substantial negative mean differences and wide limits of agreement, pointing to consistent underreporting by the SF system. Notably, the regression lines in both plots reveal a negative proportional bias, with larger underestimations occurring at higher vertical skateboard heights. This pattern suggests that the SF system increasingly underreports maximal vertical skateboard height as the actual value rises. One possible explanation is the filtering algorithm used by the SF app, which may smooth peak values more during dynamic movements. Additionally, temporal misalignment in detecting the maximal vertical skateboard height could contribute to this trend. Overall, the practical applicability of this metric is substantially limited by the magnitude of the observed errors, with MAE exceeding 20 cm for Ollies and increasing underestimation at higher jump heights. Enhancing peak detection, adjusting filter parameters, and aligning temporal markers with video-based standards could help reduce systematic deviations. It should be noted that jump-height assessments are generally challenging, as different measurement principles can lead to varying levels of accuracy [44].

Airtime measurement presented the most pronounced divergence between systems. The SF app consistently overestimated airtime compared to the OptoJump Next reference, with error metrics revealing substantial relative deviations for both Ollie and Kickflip. The paired *t*-test confirmed a statistically significant difference between the two systems, and the extremely low ICC values further indicate poor reliability across repeated trials. These findings suggest that the system’s event detection, particularly for take-off and landing, is insufficiently precise, likely due to signal smoothing or delayed threshold recognition, particularly during landing. Differences in measurement principles, such as contact-based detection in OptoJump Next versus motion-based segmentation in SF, may also contribute to the observed discrepancies. Given the relevance of airtime in trick evaluation and biomechanical analysis, improvements in temporal segmentation and signal interpretation are essential before the system can be considered valid for this metric. At the time being, using the airtime output metric of the SF for sport-specific purposes is clearly discouraged.

The observed discrepancies underscore the importance of context-aware interpretation and caution when applying the system in evaluative or comparative settings. Moreover, potential inaccuracies related to the sensors mounting location should be considered, as attaching the device directly to the skateboard might have introduced vibration-related noise, orientation shifts, or board-specific movement artefacts that could contribute to the observed discrepancies.

### 4.3. System-Level Limitations Due to Algorithmic Opacity

A limitation lies in the opacity of the SF apps’ proprietary algorithms, particularly with regard to filtering and event detection, which makes it difficult to interpret the origins of systematic biases to full extent. In addition, differences in temporal alignment between systems, for example in the definition of peaks or contact events, may have introduced further discrepancies. This opacity limits the interpretability of the observed errors, as hardware-related measurement deviations cannot be separated from algorithm-induced biases, and classification errors cannot be attributed to specific model components.

### 4.4. Methodological Limitations

Several methodological limitations to this study should be acknowledged. The study was conducted under controlled conditions with a limited sample size, which restricts the generalizability of the findings to more diverse real-world settings. Furthermore, the results depend on the chosen reference systems, such as LAVEG, Kinovea, and OptoJump Next, each of which is based on different methodological assumptions and carries its own potential sources of error, despite their established utilization of sports sciences. A minor limitation is that the LAVEG system tracks the lower back, which may exhibit forward–backward motion during pushing, whereas the foot and skateboard remain comparatively stable. This discrepancy in measurement points may introduce small deviations. Furthermore, a limitation of this work is the absence of truly negative instances within the dataset, which prevents the construction of a complete confusion matrix and inherently restricts the depth of possible classification analyses. The presence of asymmetries and extreme values in the data suggests that some distributions may deviate from normality. Although normality was confirmed by means of the Shapiro–Wilk test, where applicable, the potential influence of outliers on the parametric comparisons must be considered a limitation of this study, and the statistical inferences should therefore be interpreted with caution. Finally, the smaller analytical subsets for Kickflip classification (*n* = 12) and distance trials (*n* = 14) represent an additional limitation, as they reduce statistical power and the stability of the corresponding estimates. These constraints indicate the need for broader validation studies in the future that include larger and more diverse cohorts, balanced datasets, and explicit synchronization across systems to strengthen the robustness of the conclusions. Future research should further examine the system under standardized laboratory conditions, for example by evaluating speed measurements over predefined distances on a controlled incline or treadmill setup.

## 5. Conclusions

The results demonstrate that the system reliably detects the presence of tricks and provides reasonably accurate distance measurements. These findings support its potential for mobile performance diagnostics in skateboarding, especially in informal or training environments where flexibility and ease of use are essential. However, the limited precision of the system in terms of trick-specific classification and its substantial deviations in maximal horizontal speed, maximal vertical height of the skateboard during trick and airtime estimation highlight current technical constraints. Despite these limitations, the SF system represents a promising step toward accessible, sensor-based feedback in skateboarding. These findings may inform future applications of IMU-based feedback in skateboarding for training, coaching, and competition preparation, particularly in disciplines where objective movement analysis is currently underdeveloped. Future developments should focus on improving classification algorithms and enhancing temporal resolution to better capture the spatial and rotational complexity of skateboard tricks, including the integration of synchronization and resampling procedures required for real-time trick-recognition pipelines. Additionally, the inclusion of uncertainty metrics and adaptive calibration routines could strengthen the system’s reliability and broaden its applicability in sport science and coaching contexts. Future applications may also extend to performance diagnostics and competitive contexts, such as assessing airtime in transition skateboarding or tracking distances in halfpipe runs, thereby offering potential relevance for talent development initiatives and the evolving competitive landscape.

## Figures and Tables

**Table 1:** Overview of prior IMU-based, AI-enhanced movement and trick classification studies across acrobatic and board sports, including sensor setups, trick sets, algorithms, and reported accuracies.

Study	Sample Size	Sensor and Placement	Movement/Trick	Method/Algorithm	Accuracy/ Performance
Wesely et al., 2025 [12]	16 cheerleaders	Xsens MTw Awinda at lumbar S1	6 tumbling elements	GPC	~88–90%
Gorges et al., 2024 [13]	8 snowboarders	2 Shimmer3 IMUs on boots	Halfpipe events (take-off, airtime, landing)	1D CNN U-Net	Timing error 5–8 ms
Hu et al., 2024 [21]	9 skateboarders	Multiple IMUs	Ollie event detection (TO, HP, FL, RL)	Peak-heuristic	Timing error < 5 ms
Abdullah et al., 2021 [18]	6 skateboarders	Custom IMU behind front truck	5 tricks (Ollie, Nollie FS Shuvit, FS180, Pop Shove-it, Kickflip)	Transfer learning (MobileNet, MobileNetV2, NasNet, ResNet101/101V2) + SVM	Up to 100%
Kumar et al., 2021 [11]	4 skaters	MetaWear CPRO IMU on ankle	10 artistic skating spins	PCA + SVM, kNN, RF, DT, NB, NN; k-means	>95%; clustering 93.8–100.0%
Abdullah et al., 2020 [17]	1 skateboarder	Custom IMU behind front truck	5 tricks (Ollie, Nollie FS Shuvit, FS180, Pop Shove-it, Kickflip)	SVM, kNN, ANN, LR, RF, NB	95%
Ibrahim et al., 2020 [20]	1 skateboarder	IMU integrated into ORY board	5 tricks (Ollie, Kickflip, Shove-it, Nollie, FS180)	k-NN	85%
Corrêa et al., 2017 [16]	543 simulated signals	Artificial accelerometer signals	5 tricks (Nollie, NSHOV, Kickflip, SHOV, Ollie)	ANN (MFFNN)	98.7% (*Z* axis)
Groh et al., 2017 [19]	11 skateboarders	miPod IMMU on right front truck	11 tricks (Ollie, Nollie, Kickflip, Heelflip, BS Pop Shove-it, FS Pop Shove-it, BS 360-Shove-it, Varialflip, Hardflip, Double-Kickflip, 360-Flip)	NB, RF, Linear SVM, RBF-SVM, kNN	89.1% (correct tricks); 79.8% (all events)
Groh et al., 2015 [15]	7 skateboarders	miPod IMU behind front truck	6 tricks (Ollie, Nollie, Kickflip, Heelflip, Pop Shove-it, 360-Flip)	NB, PART, SVM, kNN	97.8%

**Table 2:** Output metrics from the SF app, description of the parameter and reference system.

Output Metrics SF App	Description	Formula	Reference System
Total distance $d_{tot}$ (m)	Horizontal travelled distance of the skateboard measured from start to end of motion sequence	$d_{tot}=x_{end}-x_{start}$	Marked line on ground
Maximal horizontal speed $v_{max}\left(t\right)$ (km h^−1^)	Highest recorded (linear) horizontal speed of the skateboard during movement	$v_{max}\left(t\right)=\frac{d}{dt}x(t)$	Laser-based ranging (LAVEG)
Maximal vertical skateboard height $h_{max}$ (cm)	Maximal elevation of the skateboard during the airborne phase defined as the highest vertical distance between the ground and the lowest point of the skateboard during airtime	$h_{max}=z_{board}(t)-z_{ground}$	2D videometry (Kinovea)
Airtime $t_{air}$ (s)	Duration between take-off and landing during a jump	$t_{air}=t_{landing}-t_{take-off}$	LED light barrier arrays (OptoJump Next)
Trick detection	Detection of a (i.e., any) trick	$=\left\{\begin{array}{c} 1,\;if\;any\;trick\;is\;identified\\ 0,\;otherwise \end{array}\right.$	Expert video analysis
Trick classification	Identification of the specific trick	Trick class ∈ {Ollie, Kickflip}	Expert video analysis

**Table 3:** Summary of valid and missing trials for each metric and corresponding practical error tolerance.

Metric	Number Participants	Trials (Valid/Missing)	Practical Error Tolerance
Total distance (m)	14	61/9	±0.5 m
Maximal horizontal speed (km h^−1^)	23	99/16	±1.0 km h^−1^
Maximal vertical skateboard height (cm)	Ollie: 23 Kickflip: 12	Ollie 56/27 Kickflip 26/16	±2 cm
Airtime (s)	Ollie: 23 Kickflip: 12	Ollie 81/2 Kickflip 37/5	±0.02 s
Trick detection	Ollie: 23 Kickflip: 12	Ollie 83/0 Kickflip 42/0	≥95% correct
Trick classification	Ollie: 23 Kickflip: 12	Ollie 83/0 Kickflip 42/0	≥90% correct

## Data Availability

Data are contained in the figures and tables within the article.
